# Synthesis of thermally robust benzimidazolone-based wholly aromatic polyketones

**DOI:** 10.1039/d0ra09831k

**Published:** 2021-01-29

**Authors:** Xinming Gu, Zhipeng Wang, Honghua Wang, Guangyuan Zhou, Yanmin Zhou

**Affiliations:** Department of Oral Implantology, Hospital of Stomatology, Jilin University Changchun 130021 China zhouym@jlu.edu.cn; Division of Energy Materials (DNL22), Dalian Institute of Chemical Physics of the Chinese Academy of Sciences Dalian 116023 China

## Abstract

A series of wholly aromatic polyketones bearing benzimidazolone moieties (PK-BI) were synthesized *via* N–C coupling polycondensation. Calcium carbonate coupled with potassium carbonate was used for the first time to achieve a high molecular weight, with *T*_g_ of the polymer as high as 299 °C. The polymer structure was confirmed by solid state ^13^C NMR and FT-IR. The thermal stability of wholly aromatic polyketones with a benzimidazolone unit in the main chain was significantly improved, being higher than those of PEEKs and other amorphous PAEKs, proved by thermogravimetric analysis (TGA) and derivative thermogravimetric (DTG) analysis. The degradation activation energy (*E*_k_) values estimated by Flynn–Wall–Ozawa (FWO) and Kissinger methods were 260.33 kJ mol^−1^ and 282.57 kJ mol^−1^, respectively, which are higher than those of PEEKs.

## Abstract 2

A series of wholly aromatic polyketones bearing benzimidazolone moieties (PK-BI) were synthesized *via* N–C coupling polycondensation.

## Graphical abstract

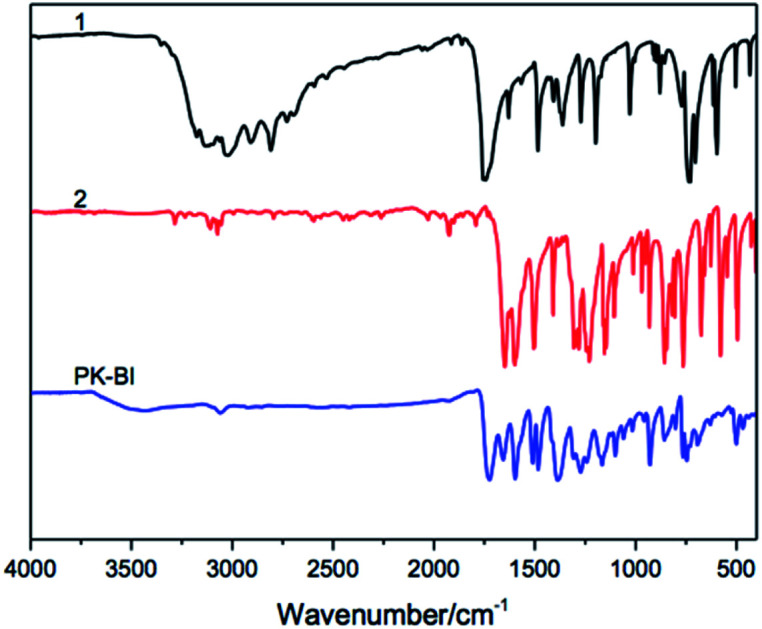

## 1. Introduction

Aromatic polymers containing only aromatic rings and ketonic carbonyl groups as dominating linkages in the main chain are defined as wholly aromatic polyketones.^1^ The structural characteristics of the wholly aromatic polyketone endowed the polymer with longer conjugating units than poly(aryl ether ketone)s (PAEKs), which are an important class of high performance resins. In the main chain of PAEK, the ether bond was a weaker linkage compared to the carbonyl group.^2^ Therefore, for wholly aromatic polyketones, the absence of ether bonds in the main chain led to a more rigid structure, and the thermal stability was enhanced. Based on the above facts, it is a good idea to prepare wholly aromatic polyketones for better thermal stability, however, the synthesis in practice encountered some inconveniences.

The difficulty in synthesis of the wholly aromatic polyketones presumably led to the paucity of reports. The reason was suspected to be its rigid skeleton caused by an aromatic ring moiety and a carbonyl moiety aligning on the same plane, which accelerated the aggregation of oligomers and precipitated in the reaction solvents even at high temperature, resulting in the suppression of the molecular weight.

**Scheme 1:**
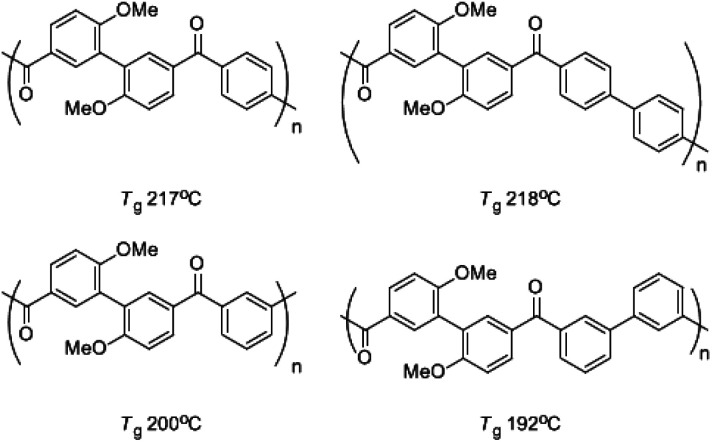
Wholly aromatic polyketones.

While some aliphatic polyketones^3^ had been synthesized, wholly aromatic polyketones,^4–12^ a new series of high-performance polymers, were reported by Yonezawa's group (see Scheme 1). There were some aliphatic substitutes such as methoxyl and trifluoromethyl side groups in these polymers synthesized by either C–C coupling polycondensation or electrophilic polycondensation considering the solubility of polymer. The glass transition temperatures (*T*_g_s) were usually lower than 220 °C. The methoxyl side groups in the polymer decreased the thermal stability of the polymer obviously compared with the aromatic backbone. Kricheldorf reported polyketones with the imidazolone unit and their poly ether ketone copolymers, but the research failed to get the wholly aromatic polyketone with benzimidazolone unit due to the low reactivity of 4,4′-diflorobenzophenone with silylated benzimidazolone in solid state melting polycondensation.^13^ Allan reported a developed N–C coupling polycondensation in TMS to synthesize the polysulfone with benzimidazolone unit.^14^ The polymer was easily soluble in chloroform and NMP.

Pyrolysis analysis was of importance in designing, processing and application of polymers. Systematic investigations about polymer characterization and mechanisms provided an efficient method to improve the structure design and achieve higher thermal durability. Features of the aromatic polymer were depicted as follows: thermal stability increased with the relative number of the aromatic groups per repeat unit of the polymer chain. The pyrolysis tended to begin with the breakage of the weakest bond in the bridging groups between aromatic rings. The introduction of the heteroatoms such as nitrogen, phosphorus and silicon by homo- or copolymerization of the monomers tended to be stable, and raised the temperature of thermal pyrolysis.

The decomposition kinetics of aliphatic polyketones based on styrene and carbon monoxide was detailed investigated by Jia's group.^15^ The *E*_k_ values estimated *via* Flynn–Wall–Ozawa method and Kissinger method were found to be in the range from 270.72 to 297.55 kJ mol^−1^. The degradation kinetics of wholly aromatic polyketone was not systematically studied yet due to the difficulty to obtain the polymer.

Here, for the first time, we reported the synthesis and the thermal degradation of a new member of heat-resistant wholly aromatic polyketone with benzimidazolone unit, PK-BI. We presumed that the big size & rigid benzimidazolone unit and nitrogen, as heteroatom, will both efficiently enhance the thermal durability compared to PEEK.

## 2. Experimental

### 2.1 Materials and apparatus

PK-BI (synthesized in our lab *via* N–C coupling polycondensation, see Section 2.2), 1,3-dihydrobenzoimidazol-2-one (1, from Alfa Aesar), 4,4′-difluorodiphenylmethanone (2, from Changzhou Huashan Chemical Co., Ltd, China), diphenylsulfone (DPS, purified prior to use), sulfolane (TMS, purified prior to use), 3-dimethyl-2-imidazolidinone (DMI, purified prior to use), 1-cyclohexyl-2-pyrrolidone (CHP, purified prior to use), anhydrous potassium carbonate and calcium carbonate (received from commercial sources and dried in vacuum at 120 °C for 24 h), poly ether ketone (PEEK, from Jilin University).

FT-IR spectrum was recorded on a Bio-Rad FTS-135 spectrophotometer. Solid state ^13^C NMR (100 MHz) spectrum was recorded on a Varian 400 instrument. Differential scanning calorimetry (DSC) was performed on a Mettler Toledo instrument DSC1 with a heating/cooling rate of 10 K min^−1^ in N_2_ (flow rate of 200 ml min^−1^). *T*_g_ was determined as the temperature at the midpoint of the thermal transition from the second heating curve. Thermogravimetric analysis (TGA) was performed on a Mettler Toledo instrument TGA/DSC1 at different heating rate from 5 K min^−1^ to 25 K min^−1^ under N_2_ or compressed air atmosphere (flow rate of 20 ml min^−1^) if the condition was not mentioned specially. *T*_−5%_ was reported as the temperature at which 5% weight loss was observed.

### 2.2 Synthesis of PK-BI

Typical procedure of preparing the polymers was described as follows: a stoichiometric ratio of monomers with an excess amount of carbonate salts (mixture of CaCO_3_ and K_2_CO_3_) was dispersed in DPS. Other aprotic solvents such as DMI, CHP and TMS were also used for comparison in our experiments. Toluene was employed to azeotrope off the water at 135 °C for 2 h, and then distilled off. The reaction mixture was stirred at 200 °C for 8 to 12 h to obtain the solution of yellowish polymer. The mixture was diluted by DMAc when the temperature decreased to 150 °C naturally. The polymer precipitated in the acidified water and ethanol. The precipitation was washed with hot acetone in a Sohlex extractor to remove the solvent, then neutralized with acetic acid and washed in boiling water to remove the salts. The product was dried in vacuum at 120 °C for 12 h.

### 2.3 Kinetic analysis of the decomposition process

Thermogravimetric analysis (TGA) was the most common technique to study the thermal degradation property of specimen. For the kinetics analysis, it was assumed that a solid to gas phase transformation during the degradation process could be in accordance with the following formula:1
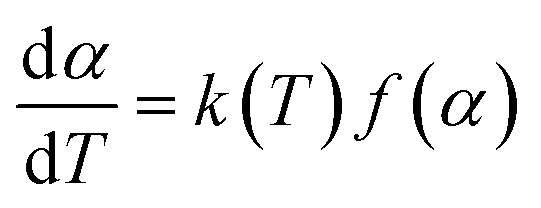
where *α* was the conversion degree defined as the ratio of the actual mass loss to the total mass loss, *k*(*T*) was the reaction rate constant, *f*(*α*) was the kinetics model function that could take various mathematical forms depending on the physical mechanism.^16,17^

When the reaction rate constant was expressed by the Arrhenius equation and taking β as the heating rate, the reaction rate could be defined as eqn (2),2
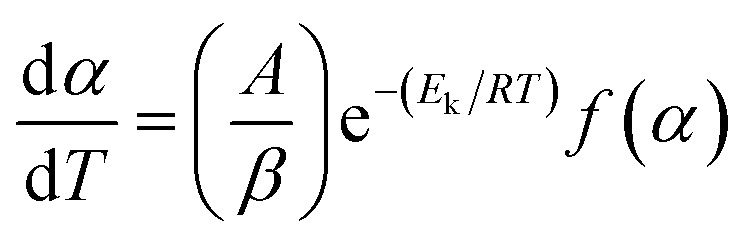


#### 2.3.1. Flynn–Wall–Ozawa method

Flynn–Wall–Ozawa method (FWO)^18^ was a simple method to determine the apparent activation energy value directly from TGA curves. After separating the variables, integrating, eqn (2) becomes:3
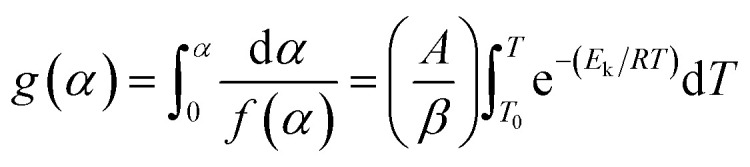


After integrating eqn (3) and taking logarithms, we obtained4
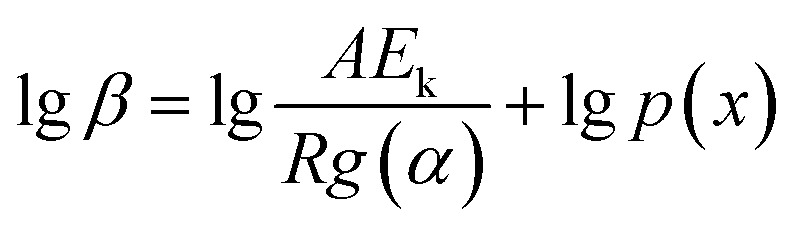
where *x* = *E*_k_/*RT*, 
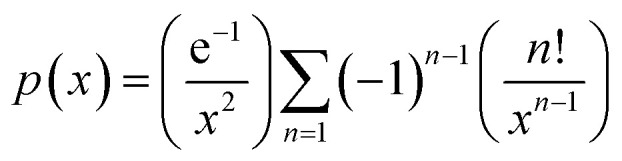
.

This function was based on the Doyle's approximation, when 20 ≤ *x* ≤ 60, lg *p*(*x*) may be closely approximated by eqn (5),5
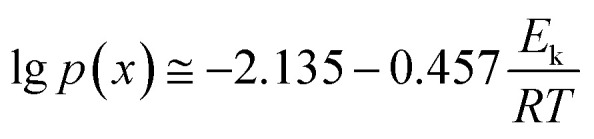


Then, eqn (4) became6
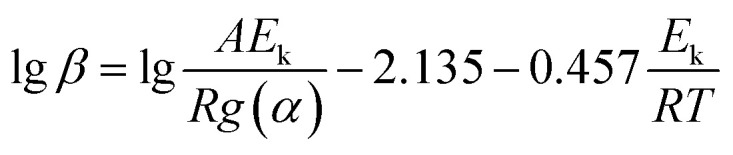


Therefore, the apparent activation energy (*E*_k_) could be obtained from the plot of lg *β* against 1/*T* for a fixed degree of conversion.

#### 2.3.2. Kissinger method

Kissinger noticed the correlation between the peak temperature (*T*_p_) and the heating rate (*β*).^19^ If we assumed the order of reaction, then the kinetic function *f*(*α*) was shown as follows:

7 *f*(*α*) = (1 − *α*)^*n*^

where *n* was the empirical reaction order. Substituting eqn (7) into eqn (2), we obtained8
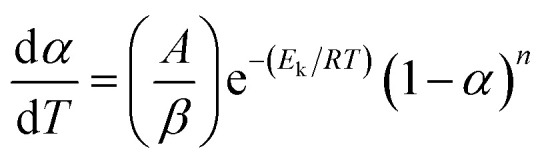


If the temperature raised at a constant rate *β*, then by differentiation of eqn (8) and defining that the maximum rate happened at the peak temperature (*T*_p_), eqn (8) became9
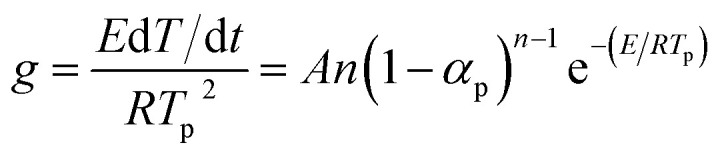


Kissinger pointed out the product *n*(1 − *α*)^*n*−1^ was very nearly equal to unity. By substituting this value in eqn (9) and taking the logarithm on the both sides, we got10
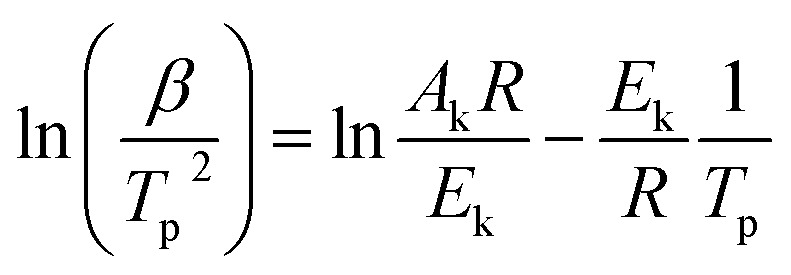


It was possible for the determination of the degradation activation energy, *E*_k_, by eqn (10). The plot of ln(*β*/*T*_p_^2^) *vs.* 1/*T*_p_ would be a straight line, the slope of which permitted the calculation of *E*_k_.

### 2.4 Pyrolysis behaviour

The pyrolysis behaviour of PK-BI was investigated on pyrolysis-gas chromatography-mass spectrometry (Py-GC/MS) in helium (He) atmosphere at 500 °C, 850 °C and 1200 °C respectively. The Py-GC/MS experiments were carried out using a CDS 5000 Pyroprobe pyrolyzer (Chemical Data System Co. Ltd) coupled to an HP 5890 gas chromatograph fitted with an Agilent 5975 mass spectrometer. The probe was calibrated by the manufacturer to ensure the accuracy of the nominally set temperatures. Sample was pyrolyzed at the setting temperature. The pyrolysis was carried out using He carrier gas at a flow rate of 50 ml min^−1^. The GC column was an HP-1MS (30 m by 0.25 mm in diameter, 0.25 mm film thickness). Oven temperature was initially held for 3 min at 60 °C, then programmed to 320 °C. The GC/MS interface was set at 250 °C. The flow rate was kept constant. The MSD was scanned from 20 to 300 *m*/*z* at a data rate of 20 Hz.

## 3. Results and discussion

### 3.1 Synthesis and characterization of PK-BI

**Scheme 2:**
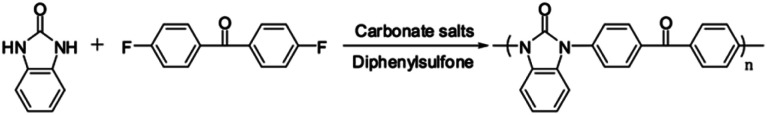
Preparation of PK-BI.

Wholly aromatic polyketone with benzimidazolone moiety (PK-BI) was synthesized *via* the N–C coupling polycondensation, from 1,3-dihydrobenzoimidazol-2-one (1) and 4,4′-difluorodiphenylmethanone (2) in the presence of excess amount of carbonate salts in DPS solvent, or DMI, CHP and TMS instead, see Scheme 2. It was indispensable to use CaCO_3_ which accomplished the leave of the fluoride group in order to get the high molecular weight polymer (Table 1).

**Table 1:** Reaction condition of wholly polyketone PK-BI containing imidazole moiety

Entry	Solvent	Base	Temperature/°C	Time/h	*T*_g_/°C
1	DMI	K_2_CO_3_	129/188	2/8	238
2	DMI	K_2_CO_3_/CaCO_3_	129/188	2/8	244
3	CHP	K_2_CO_3_	136/208	2/8	243
4	CHP	K_2_CO_3_/CaCO_3_	135/208	2/8	251
5	TMS	K_2_CO_3_	135/200	2/8	229
6	TMS	K_2_CO_3_/CaCO_3_	135/200	2/6	234
7	TMS	K_2_CO_3_/CaCO_3_	135/200	2/8	239
8	TMS	K_2_CO_3_/CaCO_3_	135/200	2/10	242
9	DPS	K_2_CO_3_	135/200	2/8	273
10	DPS	K_2_CO_3_/CaCO_3_	135/190	2/8	265
11	DPS	K_2_CO_3_/CaCO_3_	135/195	2/8	275
12	DPS	K_2_CO_3_/CaCO_3_	135/200	2/8	299
13	DPS	K_2_CO_3_/CaCO_3_	135/200	2/12	299

The effect of carbonate salts on the polymerization system was deeply investigated. The effect of calcium carbonate on the reaction could not be ignored. It was due to formation of insoluble inorganic product, calcium difluoride, with the aid of carbonate calcium. The polycondensation of monomers gave not only polymer PK-BI, but also the small molecules, such as water, carbon dioxide and potassium fluoride. With the presence of calcium carbonate, the fluoride ion would form the calcium fluoride and turn to precipitate, which lead to the accumulation of the reaction. It was revealed with the same result that a higher *T*_g_ was detected by the present of calcium salt than those without calcium salt among the four solvent DMI, CHP, TMS and DPS chosen in our experiment. We preferred DPS to other solvents including DMI, CHP and TMS in considering of the solubility of the polymer since the boiling point of DPS is high enough to ensure a high reactive temperature up to 200 °C. The precipitated calcium fluoride could be removed by washing with acetic acid, which effectively improved the efficiency of polymerization.

The effects of time and temperature on the polymerization reaction were also obvious. With the increase of reaction time and temperature, the *T*_g_ of the polymer increased obviously. However, for DPS solvent system, entry 13, no increase for *T*_g_ was observed compared to entry 12. This may be because the *T*_g_ increase was not obvious as the molecular weight of the polymer increased to a certain range, that is, when the molecular weight of the polymer reached a critical molecular weight, *T*_g_ tended to be a constant.

**Fig. 1:**
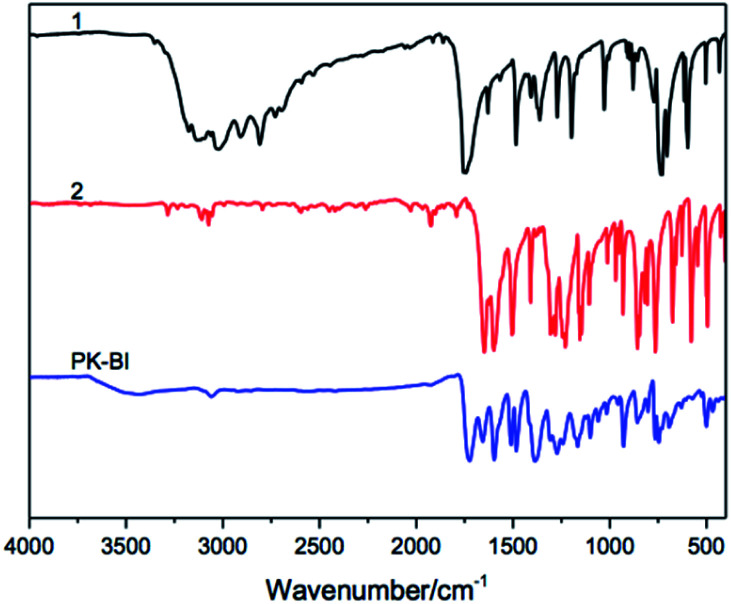
FT-IR spectra of PK-BI and the monomers.

**Fig. 2:**
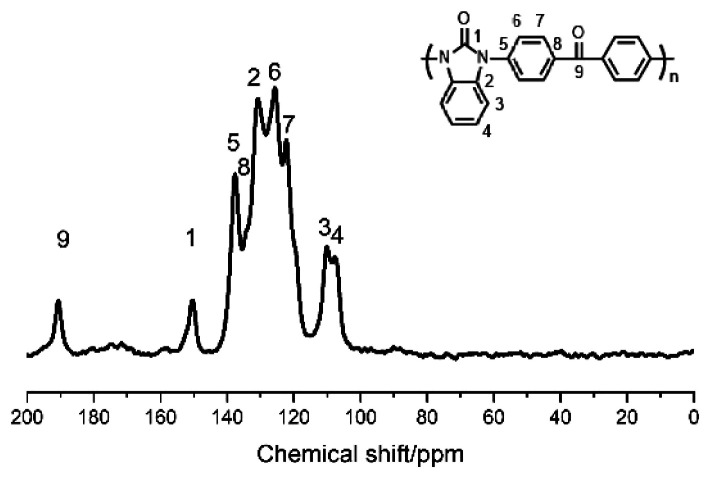
Solid state ^13^C NMR spectrum of PK-BI.

The structure of PK-BI prepared in DPS was characterized by the FT-IR (Fig. 1) and quantitative solid state ^13^C NMR (Fig. 2) spectra. The FT-IR spectra of the polymer and the monomers thereof were shown in Fig. 1. The FT-IR spectrum of PK-BI indicated that 3059 cm^−1^ was attributed to the C–H stretching vibration of the aromatic rings. The C=O stretching vibration of the urea part occurred at 1724 cm^−1^ owing to the presence of the ring strain, and that of the diphenylmethanone unit occurred at 1657 cm^−1^, 1386 cm^−1^ and 1165 cm^−1^ were attributed to the asymmetric and symmetric stretching vibration of the N–C–N group respectively.

Solid state ^13^C NMR was an effective method to characterize the structure of chemicals which were insoluble in common solvents. In Fig. 2, the signals of 190.68 ppm and 150.39 ppm were assigned to the C9 of diphenylmethanone unit and C1 of benzimidazolone moiety respectively. They were the critical peaks in the polymer. The solid state ^13^C NMR spectrum combined with the FT-IR spectrum confirmed that the structure was in accordance with that we designed.

**Fig. 3:**
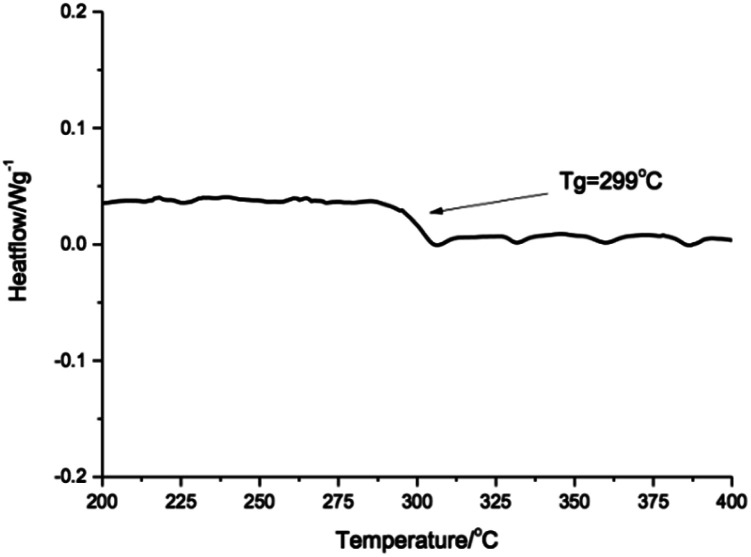
The second heating DSC thermogram of PK-BI.

Thermal property was evaluated by differential scanning calorimetry (DSC) and the second heating curve was shown in Fig. 3. There was only one glass transition at 299 °C. No melting transition was found during the test temperature range. It was in accordance with the theoretical results of the Allan S. Hay's research. The barrier to rotation of the C–O–C bond in the poly(aryl ether) resin was only 2.17 kcal mol^−1^, whereas the barrier to rotation of the C–N–C bond was calculated to be 41.79 kcal mol^−1^. The reason was that the conjugating *o*-phenylene pendent in the main polymer chain inhabited the ordered packing of the polymer. The derivative of imidazolidone unit was superior in respect to its distorting abilities, which suppressed the aggregation of the resulting polymer and maintained its solubility at the reaction temperature in DPS during the polymerization. It was necessary to note that the polymer prepared in TMS in the same condition gave a *T*_g_ of 239 °C, which was 60 °C lower than that of the polymer prepared above. This was because that the solubility of PK-BI in TMS was so poor that the oligomer would precipitate when the toluene was distilled off. The lower molecular weight of the polymer would dramatically debase the thermal and mechanical properties of the polymer. The reaction indicated that DPS was a favorable solvent in preparing polymer PK-BI.

### 3.2 Thermal degradation study

Thermal stability of PK-BI was measured by TGA. The *T*_−5%_, *T*_onset_ and *T*_p_ were determined, see Table 2. PK-BI with aromatic rings exhibited good ablation resistant property with the char yield higher than 65% at 800 °C in N_2_. It must be stressed that PK-BI showed better thermal stability than PEEK, a typical polymer in special engineering plastics at present. The probable reason for the better thermal stability was the elimination of ether linkage and the existing of C–N bond linkage in the PK-BI backbone. The bond energy of C–N was much higher than that of C–O (ether) bond.

**Table 2:** Thermal stability of PK-BI measured at different heating rate

Samples	*β*/K min^−1^	*T*_−5%_/°C	*T*_onset_/°C	*T*_p_/°C
PK-BI	5	566	554	575
PK-BI	10	578	569	591
PK-BI	15	588	580	603
PK-BI	20	594	587	610
PK-BI	25	600	593	617
PEEK	10	546	547	569

**Fig. 4:**
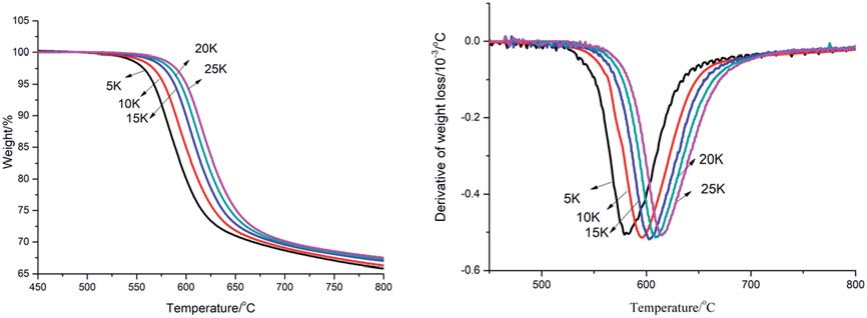
TGA and DTG curves of PK-BI with different heating rate.

PK-BI was studied by the two methods above to unveil the kinetic of the pyrogenation. The Ozawa method was reliable and suitable for the analysis of polymers without knowing the reaction mechanism.^19^ Different heating rate of PK-BI was tested. The results of heating rate at 5 K min^−1^, 10 K min^−1^, 15 K min^−1^, 20 K min^−1^ and 25 K min^−1^ were charted in Fig. 4. Since the weight loss percentage of PK-BI was higher than 65%, the activation energy was calculated from weight loss 5% to 30%.

The temperature at which the polymer weight loss happened at a certain heating rate was listed in Table 3. The apparent activation energy was calculated by the slope of lg *β* − 1/*T* at different heating rates of 10, 15 and 20 K min^−1^ according to eqn (6). The average of the apparent activation energy was 260.33 kJ mol^−1^ which was much higher than that of PEEK calculated by the same method (231.8 kJ mol^−1^).

**Table 3:** Thermal degradation active energies of PK-BI at different weight loss

Weight loss	*T*/°C					*R* ^2^	*E*_k_/kJ mol^−1^
	5 K min^−1^	10 K min^−1^	15 K min^−1^	20 K min^−1^	25 K min^−1^		
5%	566	578	588	594	600	0.9949	250.74
10%	578	590	600	606	612	0.9949	257.79
15%	588	600	610	618	634	0.9984	237.20
20%	599	610	621	628	644	0.9991	273.46
25%	614	621	638	645	651	0.9991	283.97
30%	660	638	690	696	703	0.9730	258.83

The Kissinger method was described as eqn (10). Thermal analysis of DTG showed the dynamic process when the polymer was heated. The rate of weight loss *vs.* temperature was determined. The activation energy (see Table 4) calculated by the Kissinger method from the thermogravimetric data of DTG was 282.57 kJ mol^−1^, which was higher than that calculated from FWO method.

**Table 4:** Thermal parameters and activation energies obtained from FWO and Kissinger method

*β* (K min^−1^)	*T*_p_ (K)	1000/*T* (K^−1^)	*E*_k_ (kJ mol^−1^)	
			FWO	Kissinger
10	864	1.157	260.33	282.57
15	876	1.141		
20	883	1.132		

The difference between the FWO and Kissinger methods was 22.24 kJ mol^−1^, owing to the high char yield even at the final temperature. Most of the residues which remained by the complex mechanism were nonvolatile.

**Fig. 5:**
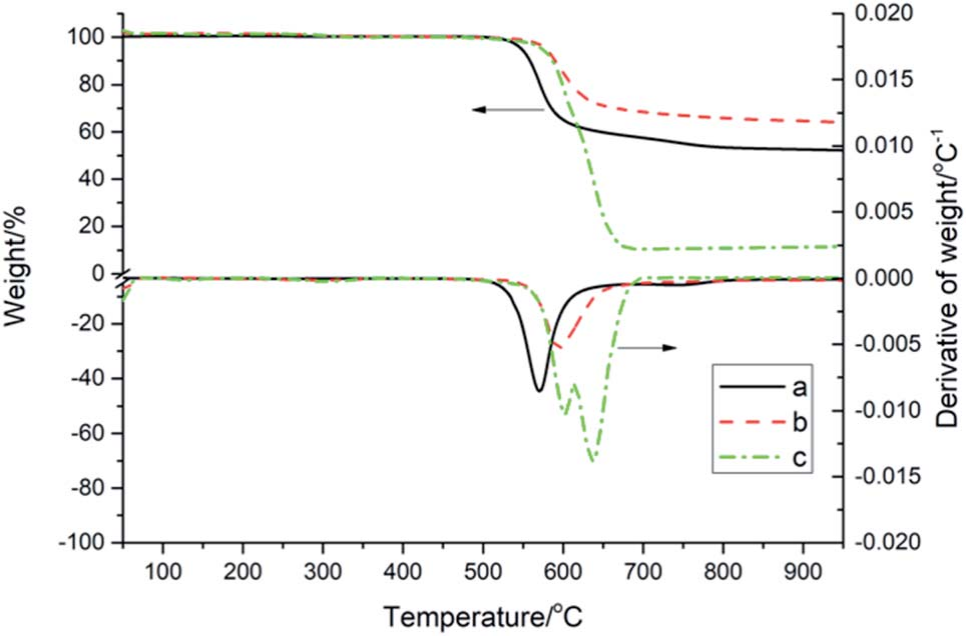
TGA and DTG curves of PK-BI and PEEK at a heating rate of 10 K min^−1^: (a) PEEK in N_2_; (b) PK-BI in N_2_; (c) PK-BI in compressed air.

The TGA and DTG curves of PK-BI in N_2_ or compressed air at a heating rate of 10 K min^−1^ were depicted in Fig. 5, and those of PEEK in N_2_ atmosphere were given for control. In N_2_, the *T*_−5%_ and *T*_p_ of PK-BI were 32 °C and 25 °C higher than those of PEEK respectively. The reason was that the present of heteroatom instead of ether linkage in PK-BI had higher bond energy than that of PEEK. The char yield values of PK-BI and PEEK in N_2_ at 900 °C were 62.0% and 52.6%. The residues of PK-BI contained more nonvolatile chemicals. The decomposition results of PK-BI in N_2_ and compressed air atmosphere were similar with each other when the temperature was below 550 °C. When the temperature increased, one peak was found in the DTG curve in N_2_ and the number for compressed air was two. The reason was that the oxygen in air reacted with the splitting aromatic free radicals. Oxygenation of aromatic rings took place and the volatile chemicals accounted for the large amount of weight loss. In compressed air, the rate of weight loss at 590 °C was twice of that in N_2_. The second peak was found at 644 °C. Much more volatile chemicals after oxygenation made the further weight loss. 68.6% of weight loss was found from 600 °C to 690 °C. The weight of nonvolatile degradation chemicals was 6.3% at 900 °C.

### 3.3 Py-GC/MS analysis

**Fig. 6:**
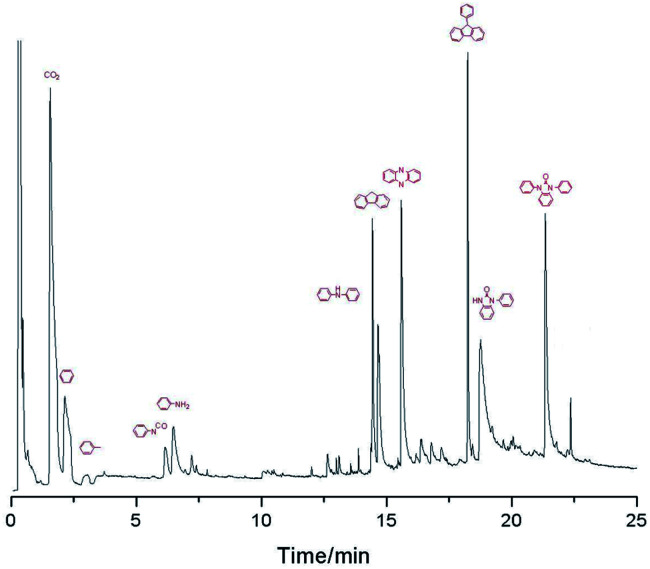
Py-GC-MS result of PK-BI at temperature of 850 °C.

Py-GC/MS was used to discuss the volatile segments decomposed of PK-BI at 500 °C, 850 °C, and 1200 °C, see Table 5 and Fig. 6.

**Table 5:** Thermal degradation products of PK-BI in He atmosphere

PK-BI		PEEK [2]	
Temp. (°C)	Chemicals detected	Temp. (°C)	Chemicals detected
500	CO_2_	450	4-Phenoxyphenol
	Toluene		1,4-Diphenoxybenzene
850	CO_2_	600	CO + CO_2_
	Benzene		Diphenyl ether
	Aniline	750	Phenol
	Fluorene		Benzene
	Diphenylamine		Dibenzofuran
	Phenazine		Hydroquinone
	9-Phenyl-9*H*-fluorene		4-Dibenzofuranol
	1-Phenyl-1*H*-benzo[*d*]imidazol-2(3*H*)-one		4-Hydroxybenzophenone
	1,3-Diphenyl-1*H*-benzo[*d*]imidazol-2(3*H*)-one		*p*-Benzoquinone
1200	CO_2_		Benzophenone
	Benzene		Biphenyl
	Aniline		Naphthalene
	Phenazine		Fluorene
	Fluorene	1100	4-Hydroxybenzophenone
	Phenazine		1,4-Diphenoxybenzene
	1-Phenyl-1*H*-benzo[*d*]imidazol-2(3*H*)-one		4-Phenylphenol
	1,3-Diphenyl-1*H*-benzo[*d*]imida-zol-2(3*H*)-one		

The results indicated that the organic decomposition chemicals were mainly the segments containing heteroatom aromatic organics and the aromatic ring such as aniline, fluorine and 9-phenyl-9*H*-fluorene. The volatile products at 500 °C were mainly carbon dioxide and toluene, indicating that the none conjugated ketonic carbonyl group was the weak bond when heated. With the test temperature ascending, 1-phenylbenzimidazolone accounting for large amounts of volatile products was detected which provided an indirect hint that the formation of C–N linkage in the reaction. The Py-GC/MS result was similar with that of PEEK^8,20^ except the heteroatom introduced by the benzimidazolone unit.

## 4. Conclusions

Wholly aromatic polyketones containing rigid benzimidazolone unit was successfully synthesized *via* N–C coupling polycondensation. Calcium carbonate as part of the catalysts, was used for the first time to achieve a high molecular weight, with *T*_g_ of the polymer as high as 299 °C. The polymer was confirmed to be highly heat-resistant due to the introducing of steric benzimidazolone unit *via* thermogravimetric analysis. The apparent activation energy of the polymer calculated by the Flynn–Wall–Ozawa method and Kissinger method was found to be higher than those of PEEKs which were the commercial special engineering plastics. The Py-GC/MS tests at different temperature indicated that the non-conjugated ketonic carbonyl group acted as the weaker bond in the main polymer chain than N–C bond when decomposition happened. The research results showed that the benzimidazolone-based aromatic polyketones remarkably enhanced the thermal stability. This prospected that the polymer had potential application in the aerospace, aviation and other fields as a super heat-resistant material.

## Conflicts of interest

The authors declare no conflict of interest.

## Supplementary Material
